# An Ecological Study of the Relations between the Recent High Suicide Rates and Economic and Demographic Factors in Japan

**DOI:** 10.2188/jea.13.56

**Published:** 2007-11-30

**Authors:** Hirokuni Aihara, Masayuki Iki

**Affiliations:** 1 Department of Public Health, Kinki University School of Medicine.

**Keywords:** high suicide rate, economic and demographic factors, Japan

## Abstract

The male suicide mortality in Japan reached the highest in 1998, since statistics were first recorded in 1899. We investigated whether the recent high suicide rates were associated with economic and demographic factors, using the Pearson’s correlation and multivariate regression analyses. The annual numbers of suicide deaths, economic variables, and the proportion of elderly people between 1995 and 2000 were obtained for all the 47 prefectures in Japan. The correlation analyses showed that the male SMR of suicide was mostly associated with the economic variables and the proportion of the elderly men. The female SMR of suicide was associated with the job application rate, and the proportion of elderly women in several years. The multivariate regression analyses showed that the male SMR of suicide was mostly associated positively with the job application rate and the proportion of elderly men and negatively with the amount of household savings and public assistance rate. The largest proportion of the variance of the male SMR of suicide was explained by the set of independent variables in 1998. The goodness-of-fit of the model concerning the female SMR of suicide was poor.

Although the number of suicides declined after the beginning of the ‘bubble economy’ in 1986, the suicide rate has gradually increased since the burst of the ‘bubble economy’ at the beginning of 1990s.^1^ In 1998 the number of suicide deaths reached 31,755 (25.3 per 100,000 people), the second highest rate after the rate (25.7 per 100,000 people) recorded in 1958.^2^^,^^3^ Characteristic was the sharp increase in the male suicide rate in 1998 (Table 1). In both 1999 and 2000 the total number of suicide deaths still remained high, exceeding 30,000.^2^ While the main reason of suicide had been ill health, this sharp increase in suicides has been suspected to be related to financial problems.^4^ In the previous studies, a downward trend in the national economy has been found to be associated with an increase in suicide rates.^5^^,^^6^ Japan has been in the mire of the recession. The average unemployment rate between 1998 and 2000 was 4.5%, while the rate had not exceeded 3.5% between 1995 and 1997.^7^ For 1998-2000 the income of workers’ households declined steadily.^8^ Three consecutive year-to-year decreases in income were first recorded for 1998-2000, since the survey was first performed in 1963.^8^

**Table 1.** National data of the economic factors, proportion of elderly people and suicide rates in Japan, 1995-2000.

	calendar year					

	1995	1996	1997	1998	1999	2000
Job application ^a^	51.3	50.7	52.8	60.5	62.8	62.3

Income ^b^ (1,000 yen)	572	585	594	593	578	565
Public assistance ^c^	13.6	13.7	13.9	14.4	15.0	15.8

Bankruptcy ^d^	68.4	66.1	74.1	84.4	67.5	81.4

Savings ^e^ (1,000 yen) % elderly ^f^	13300	13700	14100	14600	15000	15100

Male	12.0	12.5	13.0	13.5	14.0	14.5

Female	16.7	17.2	17.8	18.4	19.1	19.5

Suicide rate ^g^						
Male	23.2	24.2	25.9	36.3	36.3	35.0

Female	11.3	11.5	11.9	14.7	14.1	13.4

^a^ per 1,000 residents aged 15 years and over

^b^ Monthly average income of workers’ households

^c^ Monthly average data per 1,000 households

^d^ per 1,000 companies

^e^ per household

^f^ Proportion of people aged 65 years and over

^g^ per 100,000 residents

We investigated whether the recent high suicide rates were associated with the severe economic climate, using economic and demographic variables from the prefectural statistics.

## METHODS

Japan consists of 47 prefectures. We examined the relations between the yearly standardized mortality ratio (SMR) of suicide and economic and demographic factors. The SMR of suicide and proportion of elderly people were used to adjust the differences in age structure of the prefectures, because the yearly number of suicide deaths among age groups of each prefecture was not published. The SMR of suicide was calculated with standardization to the yearly national suicide mortality rate. The relations between suicide and economic factors (unemployment,^6^^,^^9^^-^^15^ savings,^16^ public assistance,^16^ income,^17^^,^^18^ and bankruptcy^6^) were investigated in the previous studies. In our study, the job application rate (job applicants per 1,000 residents aged 15 years and over) was used, because the yearly unemployment rate of each prefecture for 1995-2000 was not published. The number of job applicants in each prefecture was the number of those who were newly registered at the local employment security offices. The employment security offices are public facilities, where job introductions and registration for job applications are conducted. We used the monthly income of workers’ households, the amount of savings per household, the public assistance rate (the number of households receiving public assistance per 1,000 households) and the bankruptcy rate (bankrupt companies per 1,000 local companies), as economic variables indicative of the economic status of each prefecture. The Ministry of Health, Labor and Welfare, Japan, defines the public assistance system as a system which provides necessary assistance to all people who are destitute in accordance with their level of needs.^19^

The number of suicide deaths (ICD-10th; X60-84) was obtained from the yearly vital statistics of Japan.^2^ The population of the 5-year age groups was derived from the yearly Basic Register of Residents and Survey of Population.^20^ The number of job applicants in each prefecture was provided by the Department of Employment Policies of the Ministry of Health, Labor and Welfare. The numbers of bankruptcies and companies were obtained from the yearly White Paper on the National Bankrupt Companies^21^ and the yearly National Tax Agency Annual Statistics Report,^22^ respectively. The monthly income of workers’ households and amount of household savings were obtained from the Annual Report on the Family Income and Expenditure Survey^8^ and the Financial and economic statistics monthly,^23^ respectively. The data on the numbers of bankruptcies and companies, and amount of savings and income of workers’ household were shown in the yearly Minryoku.^24^ The data on the number of households, and households receiving public assistance were obtained from the yearly Basic Register of Residents and Survey of Population^20^ and Journal of Health and Welfare Statistics,^25^ respectively. The proportion of elderly people was calculated using the data derived from the yearly Basic Register of Residents and Survey of Population.^20^

The following yearly economic variables were log-transformed, because they were not normally distributed; job application and public assistance rates for 1995-2000, bankruptcy rate for 1995-97 and income of workers’ households in 1998. The yearly male and female SMRs of suicide were log-transformed, except for the female SMR in 1997. The SPSS^®^ software (version 11.0, SPSS Japan Inc., Tokyo) was used for the statistical analyses. We performed the Pearson’s correlation analysis and multivariate regression analysis using the yearly SMR of suicide and economic variables of each prefecture for 1995-2000.

## RESULTS

Table 1 shows the national data of economic variables, the proportion of the elderly people and suicide rates in 1995-2000. The remarkable increase in the male suicide rate in 1998 is noteworthy.

Table 2 shows the results of the Pearson’s correlation analyses of the relations between the SMR of suicide and economic factors and the proportion of elderly people. The male SMR of suicide was consistently associated positively with the job application rate, bankruptcy rate, and the proportion of elderly men, and negatively with the amount of savings per household in 1995-2000. An inverse relation between the male SMR of suicide and income of workers’ households was found in 1995 and 1996. A relation was found between the female SMR of suicide and the job application rate in 1997-2000. The female suicide SMR was associated with the proportion of elderly women in 1996 and 1997. The public assistance rate was associated positively with the male SMR of suicide in some years but negatively with the female SMR of suicide in 1998.

**Table 2.** Pearson’s correlation analysis of the SMRs of suicide and economic variables and the proportion of elderly people in Japan, 1995-2000 (correlation coefficients shown).

Economic factors and % of elderly	calendar year					
	1995	1996	1997	1998	1999	2000
			male			

Job-A^a^	0.48**	0.54**	0.56**	0.63**	0.59**	0.61**
Income^b^	−0.30*	−0.30*	−0.22	−0.07	−0.02	−0.18
Public-A^c^	0.24	0.33*	0.23	0.40**	0.23	0.28
Bankruptcy^d^	0.43**	0.30*	0.35*	0.56**	0.43**	0.48**
Savings^e^	−0.46**	−0.52**	−0.49**	−0.58**	−0.57**	−0.54**
% elderly^f^	0.37*	0.50**	0.44**	0.40**	0.31*	0.38**

			female			

Job-A^a^	0.13	0.25	0.36*	0.29*	0.37*	0.37*
Income^b^	0.08	−0.02	0.12	0.26	0.19	−0.04
Public-A^c^	−0.25	−0.13	−0.11	−0.31*	−0.15	0
Bankruptcy^d^	−0.02	0.01	0	−0.11	0.06	0.09
Savings^e^	0.08	0	−0.03	0.09	−0.06	−0.29
% elderly^f^	0.26	0.37*	0.38**	0.06	0.01	0.16

^a^ Job applicants per 1,000 people aged 15 years and over

^b^ per workers’ household

^c^ Public assistance rate ; per 1,000 household

^d^ Number of monthly bankruptcies divided by the number of companies

^e^ per household

^f^ Proportion of people aged 65 years and over

*p < 0.05, **p < 0.01.

Table 3 shows the results of multivariate regression analyses of the relations between the SMR of suicide and economic factors and the proportion of elderly people. The male SMR of suicide was associated positively with the job application rate for 1997-2000 and negatively with the amount of savings per household for 1995-2000. A relation between the male SMR of suicide and the proportion of the elderly men was found except in 1999. An inverse relation between the male SMR and income of workers’ household was found in 1996. The male and female SMRs were negatively associated with the public assistance rate in some years. For the years observed, the goodness-of-fit of the model was the best in 1998 when the suicide rate climbed. The female SMR of suicide was associated with the job application rate for 1997-2000 and with the proportion of the elderly women in 1996 and 1997.

**Table 3.** Regression analysis of the SMRs of suicide and economic variables and the proportion of elderly people in Japan, 1995-2000 (standardized regression coefficients shown).

Independent variable	calendar year					

	1995	1996	1997	1998	1999	2000
			male			

Job-A^a^	0.24	0.20	0.36**	0.33**	0.42**	0.37**
Income^b^	−0.08	−0.26*	−0.14	0.10	−0.06	−0.12
Public-A^c^	−0.40*	−0.23	−0.42**	−0.18	−0.39**	−0.31*
Bankruptcy^d^	0.37**	0.16	0.30*	0.34**	0.23	0.25*
Savings^e^	−0.43**	−0.44**	−0.43**	−0.48**	−0.52**	−0.46**
% elderly^f^	0.37**	0.56**	0.41**	0.27**	0.20	0.24*
R-square^g^	0.46	0.61	0.58	0.65	0.56	0.56

			female			

Job-A^a^	0.27	0.29	0.46**	0.55**	0.56**	0.46*
Income^b^	−0.07	−0.16	0.02	0.12	0.10	−0.05
Public-A^c^	−0.54*	−0.43*	−0.43*	−0.53**	−0.44**	−0.38
Bankruptcy^d^	0.12	0.10	0.07	0.02	0.06	−0.08
Savings^e^	−0.03	−0.02	−0.05	−0.01	−0.07	−0.31
% elderly^f^	0.27	0.38*	0.29*	−0.07	−0.14	0.04
R-square^g^	0.11	0.16	0.23	0.25	0.19	0.15

^a^ Job applicants per 1,000 people aged 15 years and over

^b^ per workers’ household

^c^ Public assistance rate ; per 1,000 household

^d^ Number of monthly bankruptcies divided by the number of companies

^e^ per household

^f^ Proportion of people aged 65 years and over

^g^ Adjusted R-square

*p < 0.05, **p < 0.01.

## DISCUSSION

We investigated whether the recent extremely high suicide rates were associated with economic and demographic factors. There are some limitations in our study. First, we could not use the suicide rates of specific age groups, because the yearly number of suicides among age groups of each prefecture was not obtained. The suicide rate rose after the end of the economic ‘bubble’, notably for middle-aged men.^2^^,^^26^ The current study did not reveal the relations between the suicide rates of middle-aged men and economic and demographic factors. The second limitation is ecological fallacy. The relation between the suicide rate and economic factors and the proportion of elderly people shown in our study does not necessarily suggest that the suicide deaths of individuals were associated with those factors. The third limitation is the multicolinearity in the models. Although some independent variables were correlated with each other, the correlation coefficients between the variables did not exceed 0.60, and most coefficients were below 0.50 (data not shown). The forth limitation is the bias of the information on suicide. In our study, the number of suicide deaths was obtained from the vital statistics of Japan.^2^ The data on suicide deaths can be also obtained from the White Paper on Police.^27^ Because the number of suicide deaths from the White Paper on Police is likely to be higher than that from the vital statistics of Japan, the statistics on suicide used in the present paper might underestimate the real number of suicide deaths. However, as the number of suicide deaths in each prefecture is not shown in the White Paper on Police, we obtained the data on suicides from the vital statistics of Japan.

We examined the relations between the suicide SMR and one-year lagged economic variables using the correlation analysis. The significant relations between the suicide SMR and economic factors shown in the analysis using the current economic variables were similar to those in the analysis using the one-year lagged variables (data not shown). The goodness-of-fit of the models might be more or less improved, if some one-year lagged variables are used in the multivariate regression analysis. However, we suspect that the results of the multivariate regression analysis using some one-year lagged variables differ little from those shown in the present paper.

We log-transformed some variables in the Pearson’s correlation analysis, because they were not normally distributed. The relations between the suicide SMR and economic variables and the proportion of elderly people shown in the Pearson’s correlation analysis were similar to those in the Spearman’s correlation analysis (data not shown). Therefore, we used the Pearson’s correlation analysis, because the power of the Pearson’s correlation analysis is greater than that of the Spearman’s correlation analysis.

As the demographic factors might affect the relations between the suicide and economy as confounding factors, we took into consideration population density, obtained from the Latest Demographic Statistics,^28^ and the proportion of elderly people. However, there was significant multicolinearity in the model. The two factors were significantly associated with each other (Spearman’s correlation coefficient = −0.75, p = 0). Therefore, we selected the proportion of elderly people to adjust the age structure of each prefecture.

The suicide SMR was associated with the proportion of elderly people in some years. The proportion of elderly people was associated not only with the population density but also with the proportion of the workers employed in the primary industry, obtained from the Population Census on Japan^29^ in 1995 (Pearson’s correlation coefficient = 0.75, p = 0). Thus, the proportion of elderly people might be associated with rurality. The relation between the age-adjusted suicide rate of men and rural residence was also determined by Araki et al.^30^ We suspect that the relation between the suicide SMR and the proportion of elderly people suggested that the suicide was associated with rural residence. However, the relation between the suicide SMR and the proportion of elderly people weakened for 1998-2000. The increased suicide rate in urban areas might be responsible for the weakened relation for 1998-2000.

In the previous ecological studies by Araki et al., the data on Okinawa Prefecture was excluded from the analysis.^16^^,^^30^ Although we performed the analysis of the suicide SMR, economic variables and the proportion of elderly people, excluding the data on Okinawa Prefecture, the relations between the suicide SMR and the independent variables were similar to those in the analysis including Okinawa Prefecture (data not shown).

Among the economic factors selected in our study, a relation between the SMR of suicide and job application rate was frequently found. Previous studies have reported that unemployment was responsible for suicide.^6^^,^^9^^-^^15^ However, the yearly unemployment rate of each prefecture is not published in Japan. Therefore, we used the job application rate, because we attempted to examine the relations between the recent high suicide rate and economic factors by performing the yearly analysis. A relation between suicide and bankruptcy has been shown by Weyerer S et al.^6^ Many Japanese companies have been engaged with restructuring due to the long recession^26^ and the middle-aged men are the target for restructuring.^26^ We suspect that the increase in jobless men and the failure of businesses were responsible for the sharp increase in the male suicide rate in 1998.

A few papers have examined the relation between suicide and savings. Although Araki et al. reported that no relation was found between suicide and savings in 1970 and 1975,^16^ an inverse relation was found between the male SMR of suicide and the amount of household savings. While the national unemployment rate exceeded 4.0 percent in 1998-2000, the rate had been below 2.0 percent in 1970-1975.^7^ Motohashi reported that the changes in socioeconomic structure influenced the epidemiological characteristics of suicide rates.^15^ We suspect that the effects of savings on suicide might vary, depending on the economic background.

Although the relations between income and suicide have been investigated in previous reports,^17^^,^^18^ the results have been rather conflicting. Ferrada-Noli reported a higher suicide rate in the low-income area,^17^ while Lester et al. showed higher female suicide rates in wealthier and populous areas.^18^ Three consecutive year-to-year decreases in income of workers’ household were first recorded for 1998-2000, since the survey was first performed in 1963.^8^ However, the results of the present paper suggested that income might not be a significant explanation of the suicide mortality for the time period studied. The data on the income of agricultural, forestry, fisheries workers’ households and non-workers’ households were not included in the Annual Report on the Family Income and Expenditure Survey.^8^ In addition, the data on income obtained from the Annual Report on the Family Income and Expenditure Survey^8^ was limited to the sampled household incomes in the cities where prefectural governments are located. Therefore, the income used in our study might not reflect the economic status throughout a prefecture. We used the Survey, because no other data source showing the yearly data on income for 1995-2000 were available at present. We suspect that a relation between suicide and income might vary, depending on the data source.

The public assistance rate was associated positively with the male SMR of suicide in the Pearson’s correlation analysis. However, a positive correlation between the public assistance rate and male SMR of suicide turned to be negative in the multivariate regression analysis. The inverse relation between the male SMR of suicide and public assistance rate was found when the bankruptcy rate, job application rate or amount of household savings was entered in the models. We suspect that the public assistance rate is negatively associated with suicide when the effects of some economic factors are considered.

In the present study, the proportions of the variance of the female SMR of suicide explained by the set of independent variables were small. In Japan where the primary breadwinners are men, the labor participation rates of men and women for 1995-2000 were approximately 75% and 50%, respectively.^7^ We suspect that the female suicide rate might not be strongly associated with economic factors.

The result of multivariate regression analysis revealed that the adjusted R-squares in relation to the male SMR of suicide still exceeded 0.50 for 1998-2000, even though the effects of the proportion of elderly people on suicide weakened in the yearly models. In addition, the largest proportion of the variance of the male SMR of suicide was explained by the set of independent variables in 1998, when suicide rate surged. The unemployment rate started to increase in 1998.^7^ The bankruptcy rate climbed and income of workers’ household started to decline in 1998 (Table 1).^8^ We suspect that the deterioration of economic climate was responsible for the strong relation between the male SMR of suicide and economic factors for 1998-2000. Furthermore, the present study suggests that the public assistance system might be effective in preventing suicide by providing the necessary assistance to people who are destitute in accodance with their level of needs. Job creation and expansion of unemployment security and public assistance might be needed to overcome the recent suicide crisis.
